# Evaluation of a 12-week Mediterranean diet-based nutritional and educational programme for breast cancer survivors: impact on BMI, fatigue, dietary adherence, and menopausal symptoms

**DOI:** 10.3389/fnut.2025.1629806

**Published:** 2025-08-18

**Authors:** Isabel White, Joanna Cunningham, Sofia Georgopoulou, Laura Tilt, Rachel Rawson, Ella Thilaganathan, Matthew R. D. Brown

**Affiliations:** ^1^Perci Health Ltd, London, United Kingdom; ^2^The Royal Marsden Hospital, London, United Kingdom; ^3^Field Doctor Ltd, Weston-Super-Mare, Somerset, United Kingdom; ^4^The Institute of Cancer Research, London, United Kingdom

**Keywords:** breast cancer, Mediterranean diet (MD), survivors, fatigue, digital health (DH), body mass index (BMI)

## Abstract

**Introduction:**

Breast cancer survivors commonly experience persistent symptoms after treatment. These include weight gain, fatigue, and menopausal symptoms, alongside an increased risk of long-term morbidity, including cardiovascular disease, bone loss and gut microbiome alterations. Maintaining a healthy diet is challenging due to treatment-related metabolic changes, fatigue, and dietary confusion. This research assessed the impact of a 12-week Mediterranean diet-based intervention, incorporating structured frozen meal provision and an online educational programme in breast cancer survivors. Outcomes evaluated included BMI, dietary adherence, fatigue, and menopausal symptoms. Additionally, bone, gut, and cardiovascular health within the context of survivorship was considered.

**Methods:**

A pre-post intervention evaluation design was employed. Seventy-two participants were enrolled, with 46 completing the full intervention and post-programme evaluation. The intervention comprised weekly delivery of Mediterranean diet-based frozen meals providing key nutrients. A concurrent online educational programme covered topics related to a bio-psycho-social approach to cancer rehabilitation. Primary and secondary outcomes were assessed pre-and post-intervention, educational programme engagement and adherence to meal provision were also evaluated.

**Results:**

Following the intervention, BMI decreased from 26.9 to 26.3 kg/m^2^ (*p* < 0.001). Participants classified as having a healthy BMI increased from 50% at baseline to 52.2%, while the proportion classified as overweight or obese decreased from 50% to 47.8%. Mediterranean diet adherence significantly improved, with mean MEDAS scores increasing from 6.7 at baseline to 7.9 (*p* < 0.001). The proportion of participants with high adherence to the Mediterranean diet doubled from 8.7% to 17.4%. Menopausal symptoms significantly improved, mean scores decreasing from 23.5 at baseline to 17.5 (*p* < 0.001), and the prevalence of moderate symptoms reduced from 82.6% to 63%. Fatigue levels did not change significantly (*p* = 0.37). Subjective feedback from 28% of participants indicated perceived improvements in energy levels, citing increased consumption of fiber, omega-3 fats, and fermented foods as contributing factors. Educational programme engagement varied; 89% of participants accessed at least one module, while 40% completed all modules. Adherence to the meal provision component was high, with 79% of participants consuming at least 75% of the provided meals.

**Discussion:**

The findings highlight the potential for dietary interventions to address key survivorship concerns.

## 1 Introduction

Breast cancer is the most common cancer encountered in females in the UK and is the second most commonly diagnosed cancer worldwide. As survival rates continue to improve, there's an increasing recognition of the need to address the long-term effects of treatment (1). These effects often include weight gain, fatigue, menopausal symptoms, bone density disorders and increased cardiovascular risk. All of which can negatively impact physical, emotional, and metabolic health, as well as overall quality of life (2).

Post-treatment weight gain is prevalent among breast cancer survivors, with estimates indicating that between 50 and 96% of subjects experience some degree of weight gain depending on treatment type and duration. This gain is typically attributed to dietary habits, changes in mood, hormonal changes, and reduced physical activity (3). Excess weight gain is associated with poorer outcomes, including an increased risk of recurrence and decreased survival rates (2, 4, 5). Lifestyle changes which include dietary modifications and increased physical activity are associated with better survival and quality of life (6). Tailored weight management lifestyle interventions, which promote both dietary and behavioral changes, are recommended to support long-term health in this population (7).

Cancer-related fatigue is a prevalent and debilitating symptom that affects 60% of survivors at 12 months post-diagnosis. Many patients report persistent fatigue years after completing treatment which may interfere with daily activities and reduce quality of life (8). Emerging evidence suggests that dietary factors, including adequate fiber intake, omega-3 fatty acids, and vitamin D, may influence fatigue severity (9, 10).

Menopausal symptoms, such as hot flushes, night sweats, and sleep disturbance are also frequently reported in breast cancer survivors, particularly those receiving endocrine therapy (11, 12). The use of complex carbohydrates, dietary isoflavones, omega-3 fatty acids, and vitamin D to help alleviate some of these symptoms is supported by published data (13–15).

Metabolic bone disease is another concern in patients who have received or are receiving common breast cancer treatments. Systemic anti-cancer treatments can lead to loss of bone stock and increased risk of osteoporosis and fractures (16, 17). Adequate intake of calcium, vitamin D, and other bone-supportive nutrients have been associated with improved bone mineral density, particularly in postmenopausal women (18). Lifestyle interventions promoting bone health, coupled with vitamin D and calcium supplementation, should be considered for preventing and treating osteoporosis in this population group (17, 19).

In addition to cancer-related risks, cardiovascular disease is a significant cause of morbidity and mortality in breast cancer survivors, particularly among those receiving anthracycline-based chemotherapy or aromatase inhibitors (20). Given this increased cardiovascular risk, adopting heart-healthy dietary patterns, such as the Mediterranean diet, may offer protective benefits through its emphasis on anti-inflammatory foods, fiber, and unsaturated fats (21, 22).

While observational studies have consistently linked the Mediterranean diet with improved cancer-related outcomes, few have assessed the combined impact of meal provision with an educational programme in this population (23). Existing research has largely focused on self-reported adherence to dietary recommendations, often without providing structured meal support or practical guidance (24). The logistical challenges of meal preparation, dietary confusion and treatment-related fatigue render it difficult for this population to adopt new dietary habits (25).

This work aimed to address this gap by delivering frozen ready meals designed according to Mediterranean diet principles. The meals were tailored to address common post-treatment concerns, including menopausal symptoms, fatigue and weight gain, while also encouraging longer-term dietary patterns associated with cardiovascular and bone health. Meals were delivered alongside a structured online educational programme to provide participants with practical knowledge and skills to sustain dietary changes beyond the intervention period.

The primary objective of the research was to assess changes in BMI following the 12-week intervention. Secondary objectives included assessing changes in dietary adherence, menopausal symptom severity, and fatigue levels. Additionally, while the outcomes were not directly measured, the evaluation considered the potential relevance of the programme for bone, gut, and cardiovascular health, given their importance in breast cancer survivorship and post-treatment wellbeing.

## 2 Methodology

NHS Health Research Authority guidance indicated this research does not require formal ethical approval. The study is reported in line with the Strengthening the Reporting of Observational Studies in Epidemiology (STROBE) guidelines (26). All participants received a participant information sheet (PIS) and provided written, informed consent to their participation in the research (see Supplementary materials).

This evaluation followed a pre-post design, beginning with a literature-informed meal specification development phase, followed by the online education programme development phase. Participant assessments were conducted before and after the 12-week intervention. This design was selected to evaluate changes in BMI, dietary adherence, menopausal symptoms, and fatigue within the same cohort, without the introduction of a control group.

Participants were females aged 30 to 75 years with a history of primary breast cancer (Stages I–III) who had completed primary treatment which included surgery, neoadjuvant and adjuvant chemotherapy, radiotherapy and completed or current endocrine therapy. Inclusion criteria included self-reported concerns regarding weight management, fatigue, or menopausal symptoms. Exclusion criteria included a diagnosis of metastatic breast cancer, current chemotherapy or radiotherapy, severe food allergies incompatible with the meal range, or an inability to access the online educational programme.

Participants were recruited in the United Kingdom between March and August 2024 via the Perci Health and Field Doctor networks, breast cancer support groups, and social media platforms. These networks facilitated targeted recruitment by reaching individuals seeking supportive care post-treatment. Recruitment materials included email newsletters distributed via Perci Health through relevant cancer support organizations.

### 2.1 Meal specification phase

The nutritional specification brief for the meal range was developed based on recommendations from the World Cancer Research Fund/American Institute for Cancer Research (WCRF/AICR) and the Continuous Update Project (CUP) Global reports (27). As these reports included studies published up to 31 October 2021, a literature review was conducted to identify relevant research published from 1 November 2021 to 30 September 2023. This encompassed the search terms: “*Diet, Food, and Nutrition, Micronutrients, Gastrointestinal Microbiome, Nutrition Therapy, nutritional intake, dietary or food choice and breast neoplasm, breast cancer and mortality, cancer survivorship, cancer survivors.”*

The initial search retrieved 1,593 papers 270 duplicates were removed leaving a total of 1,323 articles for title and abstract screening. After full-text assessment, 53 studies met inclusion criteria and were used to directly inform the meal design process.

Studies were included if they:

I Investigated dietary interventions in adults with a history of breast cancer.
II Examined Mediterranean diet or plant-based dietary patterns.
III Reported outcomes on BMI, fatigue, menopausal symptoms, or bone health.

Studies were excluded if they:

I Focused on pediatric populations.
II Examined patients actively undergoing cancer treatment rather than post-treatment survivorship.
III Investigated cancer types other than breast cancer.
IV Were not published in English.
V Had a sample size below 100 participants.
VI Had already been included in the CUP Global review period (pre-November 2021).

A PRISMA flow diagram detailing the literature review process is provided in the Supplementary materials.

#### 2.1.1 Findings and application to meal specification

The included studies supported the incorporation of plant-based proteins, wholegrains, fruits, and vegetables, with a specific emphasis on fiber, healthy fats, and key nutrients such as omega-3 fatty acids, vitamin D, and calcium (21–23, 28, 29). The review also highlighted the importance of dietary strategies in supporting gut microbiome diversity and cardiovascular health (30–33) to provide longer-term health benefits.

To support weight management, meals were calorie-controlled, targeting 1,500 kcal per day, based on the recommended intake for women of 2,000 calories per day, less 500 kcal per day to create a caloric deficit. Findings suggest this is effective for weight loss without compromising hunger and energy levels (34, 35).

Macronutrient distribution was aligned with UK dietary guidelines and insights from the latest scientific literature (23, 36–38). The meal composition targeted:

I 30% of total energy from fats (primarily unsaturated, including olive oil, nuts, and omega-3 sources).
II 50–55% from carbohydrates, prioritizing wholegrains, legumes, and high-fiber foods.
III 15–20% from protein, with an emphasis on plant-based sources, fish, and poultry over red meat.

Given the strong associations between diet and menopausal symptom relief, meals were enriched with soy isoflavones (via the inclusion of tofu), targeting a minimum intake of 2 mg per day, to support hormonal balance and reduce vasomotor symptoms (39–42).

To support bone health, meals were fortified with vitamin D (19, 43–46). They were designed to provide a minimum of 700 mg/day of calcium, based on UK Reference Nutrient Intake (RNI) recommendations with WCRF/AICR (27) findings supporting the need for a diet rich in calcium. The diet also incorporated magnesium, potassium, and vitamin K-rich foods to support bone density maintenance (18).

For fatigue management, the meal range incorporated:

I Higher fiber intake, which has been linked to reduced fatigue among breast cancer survivors (9, 47, 48).
II Omega-3 fatty acids from fatty fish, nuts, and seeds, which have been associated with improved energy levels and inflammatory modulation (49).
III Carotenoid-rich foods, due to emerging evidence suggesting their role in fatigue reduction and overall cancer protection (50).

The gut microbiome was another key focus, so the meal range was developed to incorporate at least 30 different plant-based foods per week, based on research indicating that higher dietary diversity is associated with greater microbial diversity (51).

The meal range excluded processed meats and limited red meat intake to three portions per week, in accordance with WCRF/AICR cancer prevention recommendations and other supporting literature (52). Nut and seed snack pots were designed to complement the meal range to support findings that consuming nuts as part of a Mediterranean diet lowers risk of heart disease related deaths, and may be associated with improved survival, particularly disease-free survival, in long-term breast cancer patients (53, 54).

Based on this evidence, the meal specification was designed to align with current dietary recommendations for breast cancer survivors and was built upon Mediterranean diet principles.

The nutritional specification brief was reviewed by key opinion leaders (senior oncology nurses and dieticians) and provided by the research team to Field Doctor's chef. Frozen ready-meals, dry porridge mixes, and nut and seed sprinkle pots were designed to meet the nutritional specification brief, with meals tailored to support weight management, menopausal symptom relief, bone health, and fatigue reduction. Further details of the nutritional specification brief are provided in the Supplementary materials.

### 2.2 Educational programme of care development phase

Educational Programme of Care modules were developed by Perci Health's clinician team comprising a breast cancer nurse specialist, weight management specialist dietitian, mindfulness practitioner and oncology exercise coach led by an oncology specialist dietician. The programme aimed to educate and support individuals after breast cancer treatment by providing evidence-based dietary and lifestyle guidance. Content aligned with the latest guidelines and with current evidence related to nutrition, exercise, weight management, and psychological wellbeing (55, 56).

The programme was structured into nine core modules, each addressing key aspects of diet, wellbeing, and lifestyle interventions post-breast cancer treatment. The content was uploaded into a programme designed by Perci Health, using a modular format to allow flexible engagement and tracking. Each module incorporated video-based education, written resources, and practical application components. Module content is outlined in Table 1.

**Table 1:** Description of nine modules in educational programme of care.

**Module**	**Module description**
1. Introduction and Goal Setting	Establishing individual health goals
2. Healthy eating after cancer and myth busting	Addressing common dietary myths (e.g., sugar, soy, dairy), WCRF recommendations, sugar, salt, saturated fats
3. Nutrition and risk reduction	Insights into the Mediterranean diet and the importance of specific nutrients for breast cancer survivors
4. Gut health	Exploring the gut microbiome's role in post-treatment health
5. Weight management strategies	Addressing post-treatment weight fluctuations
6. Exercise and physical activity	The significance of physical activity after a breast cancer diagnosis and encouraging movement and strength training
7. Psychological wellbeing and stress management	The benefits of mindfulness for mental and physical health in recovery
8. Managing key side effects	Practical strategies for dealing with fatigue, menopausal symptoms, supporting bone health, and improving sleep quality
9. Maintaining healthy habits beyond the programme	Long-term behavior change strategies

### 2.3 Intervention phase

The 12-week intervention phase comprised two components: frozen ready meal provision and online educational support.

Field Doctor was responsible for the production and delivery of meals, which were, frozen and in insulated packaging and delivered weekly to participants' homes with detailed nutritional information and instructions.

The online educational programme was delivered via Perci Health's interactive online platform, with content available on-demand, allowing participants to access the content at their convenience.

### 2.4 Adherence and outcome measures

Adherence was monitored through self-reported meal logs and module completion rates assessed via platform metrics. Participants recorded the number of intervention-provided meals consumed each week. Adherence was defined as the consumption of at least 75% of the provided meals during the 12-week intervention.

The primary outcome was the change in BMI from baseline to post-intervention. BMI was calculated using self-reported height and weight and classified according to World Health Organization (WHO) criteria.

Secondary outcomes included changes in dietary adherence, menopausal symptoms, and fatigue levels. Dietary adherence was assessed using the Mediterranean Diet Adherence Screener (MEDAS), a 14-item questionnaire assessing the frequency of key Mediterranean dietary components (57). Menopausal symptoms were evaluated using the Menopause Symptom Checklist [derived from the Greene Climacteric Scale (GCS)]. Participants rated 21 symptoms, grouped into the following subscales: psychological symptoms (11 items), somatic symptoms (7 items), vasomotor symptoms (2 items) and sexual dysfunction (1 item) (58). Fatigue levels were assessed using a validated fatigue scale, with higher scores indicating greater fatigue severity (59).

### 2.5 Statistical analysis

Statistical analyses were conducted using IBM SPSS v.29 (IBM Corp: Armonk, NY Corp). Paired *t*-tests compared pre- and post-intervention means. Pearson's correlation coefficients were calculated to explore associations between changes in dietary adherence and symptom improvement. Multiple regression analyses were conducted to control for potential confounders such as age, BMI, and treatment history. Missing data were addressed through pairwise deletion, with sensitivity analyses performed to assess potential biases. Paired *t*-tests were chosen to compare pre- and post-intervention outcomes within the same group, as the study design did not include a control group. A significance level of *p* < 0.05 was applied for all analyses.

## 3 Results

### 3.1 Participant demographics

From a pool of 119 eligible participants, a total of 85 participants completed the initial demographic survey, with 72 (84.7%) enrolling in the intervention. Of these, 46 (63.8%) participants completed the full 12-week programme and post-intervention evaluation. An additional 10 participants completed the 12-week meal and education programme but did not return the final survey, these individuals were not included in the analysis.

All participants were women aged 30 to 75 years, with a history of primary breast cancer (Stages I–III) and having completed primary treatment, including surgery, chemotherapy, and/or radiotherapy.

80.6% of participants identifying as White British. Limited representation was seen from South Asian (4.2%), Black (4.2%), and other ethnic minority groups (11%).

At baseline, the mean BMI was 26.9 kg/m^2^. Among participants completing the full programme baseline mean BMI was 27.8 kg/m^2^ with 50% having a healthy BMI (18.5–24.9 kg/m^2^), 32.5% classified as overweight (BMI 25–29.9 kg/m^2^), and 17.5% classified as obese (BMI ≥30 kg/m^2^). Results are summarized in tabular form in the Supplementary materials.

### 3.2 Educational programme engagement

Engagement with the online educational programme varied. Among the full sample (*n* = 72), 89% accessed at least one module, while 40% completed all modules. Of the 46 participants who completed the programme and post-programme evaluation, 96% accessed at least some content, but only 46% completed all modules.

Module engagement was highest for introductory content on goal setting and myth-busting (93%) but declined for later modules, particularly those addressing side effect management (59%), psychological wellbeing (59%), and physical activity (67%). The final module on long-term habit maintenance had the lowest engagement (46%).

Participants reported improved confidence in managing diet and wellbeing following the educational course, with confidence scores increasing by 20%. Post-programme surveys indicated that participants felt better equipped to navigate nutrition-related decisions and were more likely to seek reliable information from qualified professionals. Additionally, understanding of common cancer nutrition myths improved by 30%, with participants specifically mentioning increased clarity around soy, dairy, and sugar consumption.

### 3.3 Meal provision engagement

Adherence to the meal provision component was high, with 79% of participants consuming at least 75% of the provided meals during the intervention period. Participants valued the convenience of the meal provision, particularly in reducing decision fatigue and supporting dietary adherence. However, some participants found it challenging to integrate the meals into family eating routines, while others desired more flexibility in meal selection.

### 3.4 Primary and secondary outcomes

#### 3.4.1 BMI and weight management

Following the intervention, BMI decreased from 26.9 kg/m^2^ to 26.3 kg/m^2^ (*p* < 0.001). The proportion of participants classified as having a healthy BMI increased from 50% at baseline to 52.2% post-intervention, while the combined proportion of participants classified as overweight or obese decreased from 50% to 47.8%. This reduction supports other findings that suggest adherence to the Mediterranean diet may support modest but meaningful weight management improvements in breast cancer survivors. Participants with higher adherence to the Mediterranean diet experienced greater BMI reductions, further reinforcing the potential impact of dietary adherence on weight outcomes.

#### 3.4.2 Dietary adherence to Mediterranean diet principles

Mediterranean diet adherence improved following the intervention, with MEDAS scores increasing from a mean of 6.7 at baseline to 7.9 post-intervention (*p* < 0.001) reflecting a shift from lower-moderate to solid moderate adherence. The proportion of participants classified as having high adherence to the Mediterranean diet doubled from 8.7% to 17.4%. These improvements reflect increased consumption of core Mediterranean diet components, such as olive oil, nuts, legumes, fish, and wholegrains, however it falls short of the optimal 10–14 range, which is associated with the greatest health benefits.

#### 3.4.3 Menopausal symptom severity

Menopausal symptom scores decreased significantly, with the mean score reducing from 23.5 to 17.5 (*p* < 0.001). The prevalence of moderate symptoms decreased from 82.6% to 63%, with participants most commonly reporting improvements in the vasomotor symptoms (hot flushes, night sweats), and psychological symptoms (mood disturbance) subscales.

#### 3.4.4 Fatigue

Fatigue levels did not change significantly, with mean scores remaining stable throughout the intervention period (*p* = 0.37). However, individual responses varied, with 45% of participants reporting a reduction in fatigue, while 47% reported an increase. Notably, none of the participants who initially reported “extreme” fatigue maintained this level post-intervention. Additionally, three participants reported a substantial reduction in fatigue scores from 8 to 2, 8 to 3, and 7 to 1 on an 11-point numerical rating scale, suggesting that while statistical significance was not achieved, some individuals experienced meaningful improvements.

Variability in fatigue reporting was observed across different survey measures. One participant who reported a significant increase in fatigue on the post-intervention fatigue scale did not report a corresponding increase on the menopause-related fatigue question, where their responses remained stable at “a little.” This discrepancy suggests potential differences in how participants interpreted fatigue-related survey items or reflects the multifactorial nature of fatigue in breast cancer survivors.

Subjective feedback from 28% of participants indicated perceived improvements in energy levels, citing increased consumption of fiber, omega-3 fats, and fermented foods as contributing factors. However, 41% of participants did not access the module on managing fatigue, which may have contributed to the lack of a statistically significant overall improvement.

### 3.5 Qualitative feedback on the educational programme

Free-text responses provided further insight into participant experiences with the educational component. Many found the information clear and useful, particularly in dispelling common nutrition myths. However, barriers to engagement included time constraints, perceived repetition of previously known information, and a preference for live or interactive elements. Some participants suggested allowing modules to be accessed in any order, integrating a digital tracking feature, and providing downloadable reference materials to improve accessibility and engagement.

### 3.6 Qualitative feedback on the meal programme

Participants generally found the meal provision aspect highly convenient and supportive of their dietary goals. However, qualitative feedback indicated areas for improvement.

Some participants expressed a desire for greater meal variety, particularly for pescatarian and vegetarian options. Others found portion sizes too large, making it difficult to balance them with family meals. Some meals were perceived as too carbohydrate-heavy or lacking in texture, while others suggested that clearer cooking instructions and guidance on incorporating additional vegetables would enhance the experience. Concerns regarding meal packaging sustainability and food safety, particularly regarding the plastic film used on trays, were also raised.

These findings suggest that while meal provision was effective in supporting adherence, modifications to meal diversity and household integration strategies may further improve long-term acceptability and adoption.

## 4 Discussion

We explored the impact of a 12-week Mediterranean diet-based intervention, incorporating meal provision and educational content, on BMI, dietary adherence, fatigue, and menopausal symptoms in breast cancer survivors. The findings demonstrate that this structured intervention was effective in improving adherence to a Mediterranean diet, supporting weight management, and reducing menopausal symptoms, while also highlighting the complex nature of fatigue in this population. Additionally, while the intervention was not designed to measure long-term effects on cardiovascular and bone health, the inclusion of key nutrients associated with these outcomes suggests potential benefits that warrant further exploration.

### 4.1 Impact on weight management and BMI

The reduction in BMI observed in this study (from 26.9 kg/m^2^ to 26.3 kg/m^2^, *p* < 0.001) aligns with previous research demonstrating the benefits of Mediterranean dietary patterns for weight management (2, 5). Post-diagnosis weight gain is common among breast cancer survivors, with studies indicating that up to 96% of survivors experience some degree of weight gain post-treatment (3). Factors contributing to this include metabolic changes, reduced physical activity, and dietary shifts influenced by treatment side effects and psychological distress.

The calorie-controlled ready meal and snack pots were designed to prioritize fiber, plant-based proteins, and healthy fats. Research suggests that adherence to a Mediterranean diet promotes satiety, stabilizes blood glucose levels, and modulates inflammatory responses, all of which may support weight management (60, 61). However, effective weight management is not solely driven by dietary composition but also by behavioral and lifestyle factors (62).

Engagement with the weight management module in the educational programme was 78%, while engagement with the exercise module was 67%. Although the majority of participants accessed content related to dietary strategies for weight management, the lower engagement with exercise-related materials may have influenced weight-related outcomes.

Post-programme survey responses indicated that confidence in diet and nutrition strategies for weight management increased by 20%, and knowledge regarding the impact of body weight on breast cancer outcomes also improved by 20%. However, despite this increase in knowledge, qualitative feedback highlighted that some participants found it challenging to balance meals with their usual dietary habits and required additional flexibility in meal selection.

Consideration should be made as to how structured dietary support can be adapted for long-term sustainability. This may include offering more flexible meal selection options, integrating behavioral coaching on self-prepared meals, and assessing whether Mediterranean diet adherence is maintained after the intervention period. Additionally, interventions could benefit from including a more structured physical activity component, particularly given its role in maintaining metabolic health, supporting weight regulation, reducing inflammation, improving mental health, and potentially enhancing fatigue outcomes in cancer survivors (63–65). Strategies to enhance engagement with exercise guidance could include structured physical activity tracking or interactive movement-based content.

### 4.2 Impact on dietary adherence to Mediterranean diet principles

Common barriers to dietary adherence of nutrition interventions include confusion, overwhelm, misinformation, fatigue, cultural food traditions, and lack of time (66, 67). The combination of meal provision and educational support in this study appears to have addressed these challenges. The significant increase in MEDAS scores from 6.7 to 7.9 (*p* < 0.001) suggests that combining practical meal support with education is an effective strategy for reinforcing Mediterranean dietary principles.

The meal provision component minimized time-related barriers, as participants did not need to engage in extensive meal planning or preparation. Additionally, the educational programme helped combat misinformation, as evidenced by a 30% increase in participant understanding of key nutrition facts, including those related to soy, dairy and sugar. These improvements reflect an increase in dietary confidence, a key predictor of long-term adherence to dietary recommendations (66).

Despite these improvements, some participants expressed a desire for greater flexibility in meal selection and additional guidance on integrating meals into family routines. This suggests that while structured meal provision is highly effective for short-term adherence, long-term sustainability may require additional flexibility and behavioral support. Additionally, by providing meals solely for the participant, adherence may have been compromised. Gradual transition strategies, such as partial meal provision combined with self-prepared meals, may enhance long-term adherence and could be explored in future work.

### 4.3 Menopausal symptom improvement

Menopausal symptom severity significantly decreased following the intervention, with the mean score reducing from 23.5 to 17.5 (*p* < 0.001). The prevalence of moderate symptoms decreased from 82.6% to 63%, with participants most commonly reporting improvements in hot flushes, night sweats, and mood disturbances. These findings support the growing body of literature suggesting that interventions may help alleviate menopausal symptoms (14, 15). Key components included in the dietary intervention such as soy isoflavones, have been shown to exert mild oestrogenic effects, which may help modulate vasomotor symptoms such as hot flushes and night sweats (68–70). In addition, the meal range aimed to include omega-3 fatty acids which have been linked to reductions in inflammation and hormonal fluctuations, potentially contributing to improved symptom management (71). Given the high adherence to Mediterranean diet principles in this intervention, it is likely that these dietary factors played a role in the observed improvement in menopausal symptoms, which supports the existing evidence that this type of dietary pattern is associated with reductions in menopausal symptoms (72).

While the observed reductions in menopausal symptoms are promising, future studies should explore whether symptom relief is sustained beyond the intervention period. It may also be beneficial to consider how increasing engagement with physical activity modules could be beneficial for menopausal symptom management (73, 74).

### 4.4 Fatigue: the role of engagement and multi-component interventions

Despite improvements in diet adherence and weight management, fatigue levels did not change significantly (*p* = 0.37). This aligns with prior studies indicating that dietary modifications alone may be insufficient to address cancer-related fatigue, a symptom influenced by multiple factors, including inflammation, physical activity levels, sleep quality, and psychological wellbeing (75).

Individual responses, did however, vary substantially. While 45% of participants reported a reduction in fatigue, 47% reported an increase, highlighting the heterogeneity of fatigue experienced by breast cancer survivors. Notably, none of the participants who initially reported extreme fatigue maintained this level post-intervention, indicating that some individuals experienced meaningful improvements despite the lack of statistical significance within the cohort. Conversely, one participant reported a substantial increase in fatigue (from 1 to 8 post-intervention) on the fatigue scale but continued to report 'a little' fatigue on the menopause-related questionnaire. This discrepancy highlights variability in how individuals interpret and report fatigue across different measures.

Engagement with fatigue-related educational content was also variable, 41% of participants did not access the module on managing side effects, which included fatigue-specific strategies. Additionally, engagement with the physical activity (67%) and psychological wellbeing (59%) modules was lower than other modules, despite the well-established role of physical activity, stress management, and sleep optimisation in fatigue reduction (75, 76). Given the importance of multi-component interventions, the low engagement with these elements may have contributed to the lack of significant improvement in fatigue scores.

Another key consideration is the role of micronutrient status and metabolic factors in fatigue. Research suggests that glutamine deficiency, vitamin D insufficiency, and omega-3 fatty acid status may influence fatigue severity (10, 47, 77). Without biomarker assessments in this intervention, it remains unclear whether underlying nutrient deficiencies contributed to fatigue persistence. Future interventions should consider personalized supplement recommendations based on biomarker testing to determine whether targeted interventions could further support fatigue reduction in this population.

To optimize engagement and intervention effectiveness, attempts should be made to integrate structured encouragement mechanisms to increase completion rates for fatigue-related content. Strategies such as interactive features, personalized reminders, or progress tracking systems could help reinforce adherence. Additionally, incorporating guided exercise components and structured physical activity monitoring may better support fatigue management, given the well-established role of movement in reducing cancer-related fatigue.

Finally, future research should explore whether long-term adherence to a Mediterranean diet, particularly when combined with personalized nutritional and behavioral support, leads to progressive improvements in fatigue. As fatigue often persists for years post-treatment, understanding how dietary, physical activity, and psychological interventions can be sustained over time is critical for improving long-term survivorship outcomes in breast cancer survivors.

### 4.5 Longer-term health outcomes

#### 4.5.1 Bone health considerations

Bone health was a key consideration in meal design due to the well-documented risk of cancer treatment-induced bone loss in breast cancer survivors (78). This was also addressed in the educational programme, particularly through the physical activity module, which emphasized weight-bearing and resistance exercises for bone preservation (79). Engagement with this module was 67%, indicating limited exposure to bone health content for a third of participants.

As vitamin D intake can reduce fracture risk in postmenopausal women (80), future research should assess serum vitamin D status and bone-related biomarkers to evaluate the impact of dietary interventions on treatment-induced bone loss. Given the synergistic effects of diet and exercise on bone health, priority should be given to improving engagement with bone health content and supporting long-term adherence to a Mediterranean diet alongside physical activity to optimize bone mineral density (BMD) and fracture risk reduction.

#### 4.5.2 Gut health and the microbiome considerations

The potential influence of the intervention on gut health warrants further exploration, particularly given the growing recognition of the role of the gut microbiome in cancer survivorship (33, 81). Research suggests that breast cancer treatments, including chemotherapy, radiotherapy, and endocrine therapy, can alter gut microbiome diversity and composition, potentially influencing immune function, systemic inflammation, and gastrointestinal symptoms (32, 33). Dysbiosis has been observed in breast cancer survivors, with reduced microbial diversity and an imbalance in beneficial and pathogenic bacteria (82). In addition, studies suggest the estrobolome, a collection of gut bacteria involved in the metabolism and regulation of oestrogens, may play a role in modulating system estrogen levels. Disruptions in this microbial network have been linked to altered estrogen metabolism, which has been linked to breast cancer risk and recurrence (82–85).

The Mediterranean diet, which is rich in dietary fiber, polyphenols, and fermented foods, has been associated with increased microbial diversity and favorable gut health outcomes (51, 86). Prebiotic fibers from wholegrains, legumes, fruits, and vegetables encourage the growth of beneficial bacteria, and polyphenols from nuts, olive oil, and fruits such including berries have been shown to support a healthy gut microbiome (87, 88).

Adherence to the Mediterranean diet significantly improved, and confidence and understanding amongst participants improved by 30%. However, without microbiome testing, it is unclear whether this resulted in beneficial gut microbiome adaptations. Gut microbiome analysis would enable the evaluation of the long-term impact of diet & physical activity interventions on microbial composition. Understanding the relationship between dietary patterns, microbiome diversity, and systemic inflammation may provide further insights into the mechanisms underlying fatigue, hormone-related symptoms, and overall survivorship health.

#### 4.5.3 Cardiovascular health implications

Although cardiovascular outcomes were not directly assessed, cardiovascular health was a key consideration when planning the diet. Breast cancer treatments are associated with increased cardiovascular risk (20). supporting the inclusion of cardioprotective dietary strategies. The Mediterranean diet, well-established for reducing cardiovascular risk, informed the nutrition brief, emphasizing omega-3 fatty acids, polyphenols, wholegrains, and dietary fiber.

Improved Mediterranean diet adherence observed in this study suggests potential cardiovascular benefits. Participants reported greater awareness of dietary fats, favoring unsaturated over saturated fats, and described additional dietary changes, including reduced processed food intake and increased healthy fat sources. As higher fiber intake is linked to favorable lipid profiles and reduced cardiovascular risk (89), the focus on wholegrains, legumes, nuts, and vegetables may have further supported cardiovascular health.

The incorporation of cardiovascular biomarkers (e.g., lipid profiles, C-reactive protein, blood pressure) and longitudinal follow-up to evaluate the sustained impact of Mediterranean diet adherence on cardiovascular outcomes in breast cancer survivors could be explored in future work.

### 4.6 Limitations and future research directions

The limitations of this research should be carefully considered when interpreting the findings. The pre-post design without a control group limits the ability to attribute observed changes solely to the intervention, as external factors may have influenced the results. Future studies should incorporate a randomized controlled trial (RCT) design to strengthen causal inferences and better assess the direct effects of a Mediterranean diet-based intervention in breast cancer survivors. Additionally, adherence data were self-reported, which introduces the possibility of response bias, particularly in dietary intake reporting. Objective measures, such as biomarkers of dietary adherence, may enhance data accuracy in future research.

The lack of biological outcome measures, including serum vitamin D levels, gut microbiome composition, and cardiovascular biomarkers, presents another limitation. While the intervention was designed to support bone health, gut health, and cardiovascular risk reduction, the absence of direct biochemical assessments means that the physiological impact of dietary modifications could not be confirmed. Future research should incorporate biomarker assessments to determine whether dietary changes translate into measurable improvements in these health domains.

Another limitation is the lack of ethnic diversity within the participant cohort. The majority of participants (80.6%) identified as White British, with limited representation from ethnic minority groups. Given that some ethnic minorities have a higher genetic predisposition to weight gain, insulin resistance, and associated cardiometabolic comorbidities, future research should consider targeted recruitment strategies to capture a more diverse population. Recruitment approaches tailored to specific communities, such as collaborating with culturally relevant cancer support organizations, may improve inclusivity and generalisability.

Dietary preferences may have also influenced participation rates among ethnic minority groups. The Mediterranean diet, while well-supported in the literature for its health benefits, may not align with the traditional food preferences or cultural dietary patterns of certain ethnic groups. This could have been a barrier to engagement, particularly if participants perceived the intervention meals as unfamiliar or less suitable to their usual eating habits. Future interventions could adapt Mediterranean diet principles to be more culturally inclusive, incorporating staple ingredients, cooking methods, and flavor profiles that resonate with a broader range of dietary traditions while still aligning with core nutritional targets.

Furthermore, the limited ethnic representation raises questions about potential differences in dietary adherence, metabolic response, and intervention effectiveness across diverse populations. Research suggests that gut microbiome composition, response to dietary patterns, and micronutrient metabolism may vary by ethnicity (90, 91). Future studies should explore whether tailored dietary interventions—such as Mediterranean diet adaptations for South Asian, African, or Middle Eastern populations—result in comparable benefits across different ethnic groups.

Engagement with the educational programme varied, particularly for modules covering fatigue, physical activity, and long-term habit maintenance. Given that fatigue is a multifactorial issue requiring a combination of dietary, behavioral, and lifestyle interventions, limited engagement with content addressing stress management, exercise, and sleep hygiene may have contributed to the lack of significant improvements in fatigue scores. Similarly, engagement with the physical activity module was moderate, despite its relevance for both weight management and bone health. Future interventions should explore strategies to enhance engagement with key educational content, such as incorporating interactive components, structured reminders, or personalized coaching.

Future research should also investigate the long-term sustainability of dietary adherence and health outcomes beyond the 12-week intervention period. A follow-up study assessing whether participants maintain Mediterranean diet principles and whether observed improvements in BMI, menopausal symptoms, and dietary adherence persist over time would provide valuable insight into the long-term impact of structured meal provision combined with education.

## 5 Conclusion

A 12-week Mediterranean diet-based intervention, combining structured meal provision with an educational programme, led to significant improvements in BMI, dietary adherence, and menopausal symptoms in breast cancer survivors. The results highlight the potential of a Mediterranean diet to support weight management and symptom reduction in this population. However, fatigue levels did not improve significantly, which may be attributed to the multifactorial nature of cancer-related fatigue and low engagement with relevant educational content.

While the intervention successfully addressed key barriers to dietary adherence, the findings suggest that long-term sustainability and broader health outcomes, such as bone and cardiovascular health, require further investigation. Further research is needed to explore the long-term impact of dietary adherence on survivorship outcomes and to refine strategies that enhance engagement and effectiveness in this population.

## Data Availability

The datasets presented in this article are not readily available because data access restricted due to commercial sensitivity. Requests to access the datasets should be directed to matt.brown@percihealth.com.
