# Supplementary material for: Evaluation of a 12-week Mediterranean diet-based nutritional and educational programme for breast cancer survivors: impact on BMI, fatigue, dietary adherence, and menopausal symptoms
Source: Front Nutr. 2025 Aug 18;12:1629806. doi: 10.3389/fnut.2025.1629806 (PMC12400866; doi:10.3389/fnut.2025.1629806)
Supplement: Supplementary file 1 [file Data_Sheet_1.pdf]

Perci HEALTH

Diet & Wellbeing After Breast Cancer:

Developing Nutritionally Supportive Ready Meals for Women After Breast Cancer

Participant Project ID Number: (For Perci Health use) ☐

Please initial boxes you consent to

1.	I confirm that I have read and understand the project information sheet dated APR 2024 version 2.0 for the above project and have had the opportunity to ask questions.	
2.	I understand that my participation is voluntary and that I am free to withdraw at any time, without giving any reason and without my healthcare or legal rights being affected.	
3.	I agree to my completed questionnaire(s) being used for project purposes.	
4.	I understand that any personal information or project data will be anonymised to maintain confidentiality in any report or publication arising from this project.	
5.	I agree to take part in **Part 2 (work package 6)** of the above project.	

Full Name of Participant	Date	(Electronic Signature)
Full Name of Person taking consent	Date	(Electronic Signature)

1 copy for participant; 1 to be saved in WP6 sub-folder for project lead
