# Supplementary material for: Evaluation of a 12-week Mediterranean diet-based nutritional and educational programme for breast cancer survivors: impact on BMI, fatigue, dietary adherence, and menopausal symptoms
Source: Front Nutr. 2025 Aug 18;12:1629806. doi: 10.3389/fnut.2025.1629806 (PMC12400866; doi:10.3389/fnut.2025.1629806)
Supplement: Supplementary file 2 [file Data_Sheet_2.pdf]

Diet and wellbeing after breast cancer

You are being invited to take part in the development of a nutrition product that is being designed for women after breast cancer treatment. Before you decide, it's important to understand why the project is being done and what it will involve. Please take time to read the following information carefully and discuss it with others if you wish. Take time to decide whether or not you want to take part and ask us anything that is not clear.

Thank you for reading this.

What is the purpose of the project?

We know from talking to people living with breast cancer, and through published studies, that after diagnosis women often think about aspects of their lifestyle that they may want to change. This might be to better manage their health and wellbeing, side effects of treatment and for some weight management during and after treatment.

To meet this need, nutrition experts and cancer dietitians at Field Doctor Ltd and Perci Health Ltd have developed a unique collaboration that aims to provide a new frozen meal range and programme of care that supports women's recovery and wellbeing after breast cancer treatment.

Many women tell us that they want to know more about making choices around what foods to eat when they are trying to reduce their risk of cancer recurrence, deal with weight gain or treatment side effects.

While others want to change what they eat in order to improve their sense of wellbeing and to boost aspects of their physical recovery both during and after breast cancer treatment.

We want to develop and deliver an NHS recommended, specialist dietitian approved and nutritionally supportive frozen ready meal range that supports women's recovery and wellbeing after breast cancer treatment.

We are interested not only in supporting people to improve their appetite control and weight management, but to also eat food that can help to reduce the impact of treatment related effects. These might include fatigue, hot flushes and night sweats and improve bone health and foods that may reduce the risk of cancer recurrence.

This project also provides a 12 week meal plan and educational programme making it easier for those living beyond a breast cancer diagnosis to eat a diet that meets the World Cancer Research Fund 2018 recommendations on reducing cancer risk.

We also provide you with the science behind some common dietary myths so that you have more confidence in what you are eating.

Why have I been chosen?

You have been asked to take part in this project because you have been treated for breast cancer and we believe that you have important insights and information about changes you may have made or plan to make to your diet. These might be based on what you have read, seen in the media or been told by health professionals or others treated for breast cancer.

You have been identified to take part in this project because you are over 18 years of age and have either completed your treatment for breast cancer or are taking hormone (endocrine) therapy. You may be interested in the relationship between what you eat and how this can be modified to enhance your recovery after cancer and reduce overall risk of cancer recurrence.

Do I have to take part?

It is up to you to decide whether or not to take part. If you do decide to take part, you will be given this information sheet to keep and be asked to sign a consent form sent to you by email. If you decide to take part, you are still free to withdraw from the project at any time without giving a reason.

A decision to withdraw at any time, or a decision not to take part, will not affect the type or quality of care and support you receive. However, if you decide not to take part it would be very helpful to us to know the reason for your decision. This may help us in the management and design of future projects on this topic.

What will happen to me if I take part?

If you agree to take part in this project, we will ask you to complete some questionnaires and possibly a short interview to capture information you wish to share with us about the dietary challenges that you may have faced after your breast cancer diagnosis and treatment.

A member of the Perci Health project team will contact you by email within 1-3 weeks after reviewing your completed consent form and demographic details questionnaire. This email contact will inform you if the project is suitable for you to take part in, to return a copy of your completed consent form to keep for your records and to send you the necessary project questionnaire(s) to complete and return to us.

A team member will also be available to assist you, as needed, with any aspect of filling in any demographic details or project survey questionnaires used and it's not expected to take any longer than approx. 30 minutes to complete.

What do I have to do?

If you decide to take part in this project you don't have to change any aspect of your lifestyle, medication, care or follow up. What we ask is that you are willing to answer the questions asked in a brief interview and questionnaires in as full and honest a manner as you can.

The overall project has seven work packages, takes place over an 18-20 month period and started in July 2023.
Part 1 / Work Package 4 of the project- Building our nutritional programme of care for women after breast cancer treatment- ran from March-April 2024.
We recruited 10-15 women who had completed their acute treatment for breast cancer, some of whom were still receiving hormone (endocrine) therapy (Tamoxifen, Letrozole, Zoladex etc.) and 4 key opinion leaders (specialist oncology dieticians & breast care specialist nurses) to give us feedback on the proposed modules and outline content for our a nutritional programme of care. This information was used to shape and develop the nutritional programme of care that you are now able to take part in work package 6

The work package with which we would like your help is:

Work Package 6- Testing our new Field Doctor / Perci Health meal range & nutritional programme of care

In work package 6 (WP6) you are offered the opportunity to test and give feedback on our new frozen meal range and 12 week meal plan created by this unique partnership between Field Doctor and Perci Health so we can make sure that this product range meets your needs, is tasty and enjoyable to eat and can be part of your busy work and home lives whilst being suitable for personal, cultural or religious observances.
You will also be asked to complete our 12 week nutritional programme of care designed to support you to live and eat well after breast cancer

If you decide to take part in **project work package 6** you will be asked to:

- Complete a written consent form and brief demographic details questionnaire that tells us something about you, your diagnosis and treatment (**if you did not complete these documents already as part of WP4**)

- Complete up to six questionnaires before the start and on completion of your 12 week meal plan and programme of care that capture information about the changes you might experience as a result of taking part in this project

- Try out our full range of specially created frozen meals created by Field Doctor, in your own home, as a key component (minimum of one meal per day) of your overall diet for a 12 week period

- Take part in a brief email survey questionnaire (no more than 20 questions) to give us feedback on your views of the Field Doctor meal range.

- Complete our newly designed nutritional programme of care developed by clinical experts in nutrition, diet and physical activity after cancer based on published literature

- Answer a brief email survey questionnaire (no more than 20 questions) about what you found most useful about our nutrition programme of care and whether or not it met your information and support needs related to diet and nutrition after breast cancer treatment

Our 12 module (completed over a 12 week period) nutrition programme of care includes the following:

- Offers you one module per week designed to help you pace yourself and support you in the dietary changes you want to make

- A recipe bank to help you make healthy meals designed specifically to support your recovery, help you with weight management and reduce cancer-related fatigue

- Brief prompts and questions that help you to achieve your personal diet or nutrition goals

- Information and support related to physical activity, emotional well-being and stress reduction, improving sleep and dietary strategies for symptom management

All information you share with us will be stored in our password protected project database and when used for project reporting or publication purposes, your personal details will be removed so that you, as an individual, cannot be identified from the information you have given us.

What are the possible disadvantages and risks of taking part?

We do not anticipate that there are any risks associated with taking part in this project. If it does raise any issues that you would like to discuss further, we are happy to provide information or suggest other specialist sources of advice. However, if you feel there are other things that may affect your experience, such as other illnesses, please feel free to discuss this with a member of the project team prior to taking part.

What are the possible benefits of taking part?

This project may benefit you in a number of ways. We believe that the information gained from our 12-week nutritional programme of care will help to improve your knowledge and confidence to select the food and drinks most likely to support you to improve your wellbeing and recovery after breast cancer treatment.

What if something goes wrong?

We believe that this project is safe and do not expect you to experience any harm or injury because of your participation in it.

If you are dissatisfied about any aspect of your involvement in this project you have the right to complain to: Rachel Rawson (Innovate Project Lead & Perci Health Lead Clinical Nurse Specialist) by email at: **eatingwell@percihealth.com** Participation in this project will in no way affect your legal rights.

Will my taking part in this project be kept confidential?

Only the project team at Perci Health will have access to the completed questionnaire booklets and consent forms and these will be stored securely on password protected computers used by the Perci Health Project Team. All information regarding your medical history that you have shared with us will be treated in strict confidence.

For this project, relevant personal details such as your age and menopausal status, your height and weight, breast cancer diagnosis and treatment will be recorded and stored. You will be asked to give permission for us to record these details for project purposes. Your name and other identifying details will be removed and will not be used on any reports that are produced from the project.

In the unlikely event that, through your project involvement, you disclose that you or someone else may be at risk, confidentiality may be broken. In this instance, the project lead would act in your best interest by informing relevant services to provide appropriate support or assistance.

What will happen to the data?

We need to provide you with information about how your data are processed for transparency purposes under the new General Data Protection Regulation, or GDPR as it is more commonly known.

Perci Health Ltd and Field Doctor Ltd are collaborators on this project based in the United Kingdom. We will be using information from you in order to undertake the project and will act as the data controller for this project. This means that we are responsible for looking after your information and using it properly. Perci Health Ltd will keep identifiable information about you for at least five years after the project has finished, in line with local

policies and legal requirements. You can find out more about how Perci Health and Field Doctor Ltd uses your information by contacting the Data Protection Officer at:
Data Protection Officer - Field Doctor: alex@fielddoctor.co.uk
Data Protection Officer - Perci Health: dpo.percihealth@kdpc.uk

Certain individuals from Perci Health orField Doctor and regulatory organisations may look at your project records to check the accuracy of the project data held. The people who analyse this information will not be able to identify you and will not be able to find out your name or contact details.

Your rights to access, change or move your information are limited, as we need to manage your information in specific ways in order for the project data collected to be reliable and accurate. If you withdraw from the project, we will keep the information about you that we have already obtained. To safeguard your rights, we will use the minimum personally identifiable information possible.

What will happen to the results of this Field Doctor and Perci Health project?

The results of this project will be published in a report to the grant awarding panel at Innovate UK.

Innovate UK is the UK's national innovation agency. It supports business led innovation in all sectors, technologies and UK regions. Innovate UK helps businesses improve the development and delivery of new products, processes and services.
Innovate UK is also part of the UK Research and Innovation (UKRI) national funding agency that invests in science and research in the UK. UKRI is an executive non departmental public body, sponsored by the government's department for science, innovation and technology.

It's also anticipated that the project report will be summarised for publication in a suitable professional journal to share the results of this important project with other cancer health care providers supporting women after breast cancer treatment.

Who is organising and funding the project?

This project is funded by a project grant from Innovate UK and is organised by a team from Field Doctor Ltd.
The project lead overseeing the conduct of this project at Perci Health Ltd is: Rachel Rawson, Lead Clinical Nurse Specialist (CNS).

Who has reviewed the project?

This project has been reviewed by the Innovate UK grant awarding panel. It has also been peer reviewed by experts in cancer and nutrition as part of the Innovate UK project application review process.

Contact for further information

You will always be able to contact a project team member to answer any questions you may have, to discuss any concerns and/or to get advice:

Name: Rachel Rawson
Designation: Project Lead & Perci Health Lead Clinical Nurse Specialist (CNS)
Place of Work: Perci Health Ltd.
Email: eatingwell@percihealth.com

Thank you for considering taking part in this project.
