# Supplementary figures and images for: Evaluation of a 12-week Mediterranean diet-based nutritional and educational programme for breast cancer survivors: impact on BMI, fatigue, dietary adherence, and menopausal symptoms
Source: Front Nutr. 2025 Aug 18;12:1629806. doi: 10.3389/fnut.2025.1629806 (PMC12400866; doi:10.3389/fnut.2025.1629806)

PRISMA Flow Summary for Literature Review (1 Nov 2021 – 30 Sept 2023)

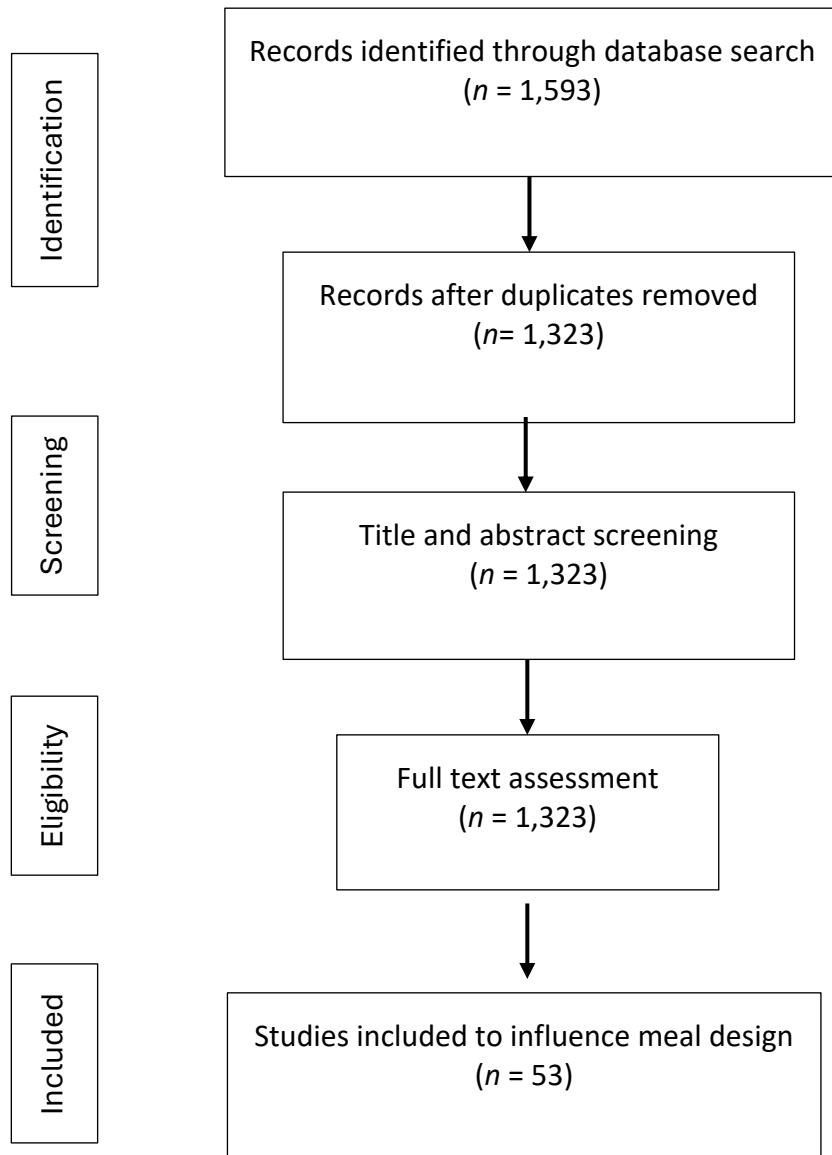

Supplement: Supplementary file 3 [file Table_1.pdf]
