# Supplementary material for: Evaluation of a 12-week Mediterranean diet-based nutritional and educational programme for breast cancer survivors: impact on BMI, fatigue, dietary adherence, and menopausal symptoms
Source: Front Nutr. 2025 Aug 18;12:1629806. doi: 10.3389/fnut.2025.1629806 (PMC12400866; doi:10.3389/fnut.2025.1629806)
Supplement: Supplementary file 4 [file Table_2.pdf]

Nutritional Specification Brief

RANGE

Breast Cancer Meal Plan

TARGET MARKET

A range of ready-meals tailored towards people living beyond a breast cancer diagnosis, based on the best available evidence.

Specific dietary needs focus on:

- Recurrence risk reduction
- Weight loss / improvements in body composition / appetite control
- Supporting bone health
- Minimising fatigue
- Supporting menopausal symptoms (e.g. vasomotor symptoms)

Output:
Develop a whole weekly meal plan - to include breakfast, main meal and snack options.

CUSTOMER PROFILE

Female
Age >18
Completed cancer treatment
Possibly experiencing residual effects from treatment

Key topics of concern for this group:

- Weight
- Menopause symptoms
- Fatigue
- Fears around dairy, soy, red meat & sugar

DAILY / WEEKLY TARGETS, KEY CONSIDERATIONS & EXCLUSIONS

**Daily Macronutrients**		**Carbohydrates**	**Protein**	**Fat**
	**% of energy**	**50%**	**20%**	**30%**
	**Kcal**	**g per day**	**g per day**	**g per day**
Breakfast	300	38	15	10
Lunch	450	56	23	15
Dinner	450	56	23	15
Drinks/snacks	300	38	15	10
**Total per day**	**1500**	**188**	**75**	**50**

Nutrient/Food	Daily Target	Notes
Fibre	30g	Include whole grains, non-starchy vegetables, fruits, and pulses (legumes) such as beans and lentils<br><br>30 total plants per week<br><br>Include 5-9 servings (at least 400 g in total) of a variety of non-starchy vegetables and fruits every day:<br>- 3 fruit<br>- 3+ vegetables<br>- 3 wholegrains & starchy vegetables<br>- legumes x3 per week (include all types; kidney & edamame - omega3)<br>- nuts x3 per week<br>- seeds: include flax, chia & hemp (omega3)<br><br>Choose low GI options where possible<br><br>Include sources of carotenoids<br><br>Include diversity within each vegetable category i.e. different colour peppers, different mushroom types, different leafy greens
Calcium	1000-1200mg	From plant-based sources & low fat dairy<br><br>*Note: spinach & other foods containing oxalates inhibit the absorption of calcium*
Vitamin D	400IU	Food sources to be included as much as possible<br><br>*(WP4 will provide supplement recommendation to meet daily targets as this is unlikely to be possible through food)*
Vitamin K	90mcg	
Isoflavone from soy foods	2mg	
Extra Virgin Olive Oil	1-4 tablespoons	

Food	Weekly Target	Notes
Fish	3 servings	High in Omega-3s
Legumes	3 servings	
Nuts	3 servings	Including walnuts, almonds & hazelnuts

Key Considerations:	Target	Notes

Low in saturated fat	< 1.5g per 100g	< 20g per day
Low in salt	< 1.5g per 100g	< 6g per day
Low in sugar	< 5g per 100g	
Rich in polyphenols	N/A	Fruit & Veg<br><br>Herbs & Spices (e.g. sage, thyme, marjoram, parsley, rosemary, basil, bay, cloves, caraway, cumin, ginger, nutmeg, star anise, vinegar, soy sauce, cinnamon, pepper)

**Exclusions:**
NO alcohol
NO processed meat
NO grapefruit or grapefruit juice
