# Supplementary material for: Evaluation of a 12-week Mediterranean diet-based nutritional and educational programme for breast cancer survivors: impact on BMI, fatigue, dietary adherence, and menopausal symptoms
Source: Front Nutr. 2025 Aug 18;12:1629806. doi: 10.3389/fnut.2025.1629806 (PMC12400866; doi:10.3389/fnut.2025.1629806)
Supplement: Supplementary file 5 [file Table_3.pdf]

Results Table

Parameter	Result
Age 51-60 Years	*n* = 20
Age 41-50 Years	*n* = 17
Age 30-40 Years	*n* = 4
Age 61-70 Years	*n* = 4
Age Over 70 Years	*n* = 1
Oncosurgical treatment	97.8%
Chemotherapy	45.7%
Radiotherapy	78.3%
Endocrine treatment	76.1%
Pre-post Body Mass Index (BMI)	26.9 kg/m^2 to 26.3 kg/m^2 ($p < 0.001$)
Pre-post fatigue score (11 point numerical rating scale)	4.98/10 to 5.11/10 ($p = 0.37$)
Pre-post Menopausal Symptom Score	23.5 to 17.5 ($p < 0.001$)
Pre-post Mediterranean Diet Adherence Score (MEDAS)	6.7 to 7.9 ($p < 0.001$)
